# Pro- and anti-inflammatory macrophages express a sub-type specific purinergic receptor profile

**DOI:** 10.1007/s11302-021-09798-3

**Published:** 2021-07-20

**Authors:** J. Merz, A. Nettesheim, S. von Garlen, P. Albrecht, B. S. Saller, J. Engelmann, L. Hertle, I. Schäfer, D. Dimanski, S. König, L. Karnbrock, K. Bulatova, A. Peikert, N. Hoppe, I. Hilgendorf, C. von zur Mühlen, D. Wolf, O. Groß, C. Bode, A. Zirlik, P. Stachon

**Affiliations:** 1 Department of Cardiology and Angiology I, Heart Center Freiburg University, Faculty of Medicine, University of Freiburg, Hugstetter Str. 55, 79106 Freiburg, Germany; 2 Institute of Neuropathology, Medical Center - University of Freiburg, Faculty of Medicine, University of Freiburg, 79106 Freiburg, Germany; 3 Faculty of Biology, University of Freiburg, 79104 Freiburg, Germany; 4 Signalling Research Centres BIOSS and CIBSS, University of Freiburg, 79104 Freiburg, Germany; 5 Center for Basics in NeuroModulation (NeuroModulBasics), Faculty of Medicine, University of Freiburg, 79106 Freiburg, Germany; 6 Department of Cardiology, University Hospital Graz, Graz, Austria

**Keywords:** Macrophages, Polarization, Purinergic receptor: Inflammation

## Abstract

Extracellular nucleotides act as danger signals that orchestrate inflammation by purinergic receptor activation. The expression pattern of different purinergic receptors may correlate with a pro- or anti-inflammatory phenotype. Macrophages function as pro-inflammatory M1 macrophages (M1) or anti-inflammatory M2 macrophages (M2). The present study found that murine bone marrow-derived macrophages express a unique purinergic receptor profile during in vitro polarization. As assessed by real-time polymerase chain reaction (PCR), Gαs-coupled P1 receptors A2A and A2B are upregulated in M1 and M2 compared to M0, but A2A 15 times higher in M1. The ionotropic P2 receptor P2X_5_ is selectively upregulated in M1- and M2-polarized macrophages. P2X_7_ is temporarily expressed in M1 macrophages. Metabotropic P2Y receptors showed a distinct expression profile in M1 and M2-polarized macrophages: Gαq coupled P2Y_1_ and P2Y_6_ are exclusively upregulated in M2, whereas Gαi P2Y_13_ and P2Y_14_ are overexpressed in M1. This consequently leads to functional differences between M1 and M2 in response to adenosine di-phosphate stimulation (ADP): In contrast to M1, M2 showed increased cytoplasmatic calcium after ADP stimulation. In the present study we show that bone marrow-derived macrophages express a unique repertoire of purinergic receptors. We show for the first time that the repertoire of purinergic receptors is highly flexible and quickly adapts upon pro- and anti-inflammatory macrophage differentiation with functional consequences to nucleotide stimulation.

## Introduction

Macrophages play a fundamental role in many immunological processes and orchestrate inflammation in many diseases [1]. Their immunological impact is highly flexible and dependent on environmental stimuli of the current surrounding. According to their immunological function, macrophages can be divided into a pro-inflammatory M1 and an anti-inflammatory M2 subtype. In this context, pro-inflammatory stimuli like interferon (IFNγ) and lipopolysaccharide (LPS) promote the M1, whereas stimuli like interleukin-4 (IL-4) promote the anti-inflammatory M2 polarization [2]. A detailed characterization of this polarization spectrum is challenging and valid surface markers for M1 and M2 differentiation remain to be discovered [3].

Several stimuli within the extracellular matrix promote macrophages towards an anti- or pro-inflammatory phenotype. One important pro-inflammatory factor is extracellular nucleotides and their recognition by purinergic receptors. Dying cells release nucleotides into the extracellular environment where they act as so-called damage-associated molecular patterns (DAMPs). There, nucleotide triphosphates such as ATP and UTP can be de-phosphorylated by the ectonucleotidase CD39 to nucleotide diphosphates like ADP and UDP as well as inert nucleotide monophosphates such as AMP and UMP. The ectonucleotidase CD73 converts nucleoside monophosphates (AMP and UMP) to nucleosides such as adenosine and the inert uridine. Thus, ectonucleotidase-dependent degradation shapes the composition of extracellular nucleotides. This extracellular nucleotide and nucleoside composition orchestrates immunological processes via the activation of purinergic receptors. Purinergic receptors can be divided into adenosine recognizing P1 receptors and nucleotide recognizing P2 receptors. G-protein coupled P1 receptors have rather anti-inflammatory effects, whereas metabotropic G-protein coupled P2Y and ionotropic ATP-dependent P2X receptors are rather pro-inflammatory. P1 receptors are further sub-divided into high sensitivity Gαi (A1) and Gαs (A2A) as well as low sensitivity Gαi (A3) and Gαs (A2B) receptors. Murine nucleotide P2Y receptors can be divided into the Gαq-coupled ADP-dependent P2Y_1_, ATP-dependent P2Y_2_, ATP/UTP-dependent P2Y_4_, and UDP-dependent P2Y_6_ as well as the Gαs-coupled ADP-dependent P2Y_12_, P2Y_13_ and UDP-glucose-dependent P2Y_14_ receptor. The ionotropic P2X receptors P2X_1-7_ are all activated by ATP and differ in open-time, ion-flux, and ATP sensitivity with P2X_7_ building up the biggest pore, the most pronounced ion-flux and lowest sensitivity to extracellular ATP [4]. It is known that certain cell types like neutrophils express a unique profile of purinergic receptors [5]. This unique receptor profile integrates into a specified reaction to extracellular nucleotides/nucleosides. For instance, activation of the formyl-peptide receptor by pathogen-derived formylated peptides promotes the release of ATP and thereby activates adjacent P2Y_2_ receptors. This pathogen-directed signal leads to the translocation of A3 receptors to the side of P2Y_2_ activation. Membrane-bound ectonucleotidases hydrolyze ATP and produce an apico-lateral adenosine gradient activating the translocated A3 receptor. This apical A3/P2Y_2_ composition promotes chemotaxis of neutrophils towards an formylated peptide-driven ATP gradient [5]. Accordingly, the expression of a certain purinergic receptor profile further enhances the complexity of extracellular nucleotide/nucleoside signaling and shapes environmental stimuli.

The purinergic receptor profile uniquely defines the cellular behavior to nucleotides and nucleosides in the extracellular environment. In this work we aim to analyze the purinergic receptor profile of pro-inflammatory and anti-inflammatory macrophages. We further want to access if this profile correlates with molecular behaviors in these macrophage subtypes and possibly serve as potential new M1/M2 marker.

## Materials und methods

### Macrophage cultivation and differentiation

C57Bl6/J mice were euthanized using CO_2_. Both femurs were collected; the diaphysis removed and rinsed though a 40-μm cell strainer (Falcon) with ice-cold PBS w/o calcium and magnesium (Lonza). These bone marrow cells were centrifuged with 300*g* for 5min at 4°C, re-suspended in macrophage differentiation medium containing 500ml DMEM (Gibco), 10% FCS (PAN Biotech), 1% penicillin-streptomycin (P/S) (Bio Whittaker), and 30ng/ml M-CSF (PeproTech) and plated in an incubator at 37°C and 5% CO_2_. Non-adherent cells were removed after 3 days by replacing the macrophage differentiation medium. The macrophage differentiation medium was removed after 7 days, and adherent cells were washed twice with PBS with calcium and magnesium (Lonza). Macrophage differentiation and cell culture purity was verified via flow cytometric analysis. For M1 differentiation, BMDMs were incubated 5h at 37°C and 5% CO_2_ with DMEM + 10% FCS + 1% P/S with 10ng/ml IFNγ (PeproTech) and 100ng/ml LPS from *Escherichia coli* 055:B5 (Sigma Aldrich). For M2 differentiation, BMDMs were incubated 5h at 37°C and 5% CO_2_ with DMEM + 10% FCS + 1% P/S with 20ng/ml IL-4 (PeproTech). Non-stimulated M0 macrophages only received DMEM + 10% FCS + 1% P/S for 5h at 37°C and 5% CO_2_.

### Flow cytometry

Bone marrow cells incubated for 7 days with macrophage differentiation medium were rinsed twice with PBS w/o calcium and magnesium (Lonza). BMDMs were detached using 5 mM EDTA (AppliChem) in PBS w/o calcium and magnesium. Detached cells were centrifuged at 300*g* for 5min and re-suspended in FACS buffer (PBS w/o calcium and magnesium + 1% FCS + 0.5% BSA (AppliChem)). Lineage staining was introduced by adding CD3e (clone: 145-2C11, eBioscience), CD19 (clone: eBio1D3, eBioscience), NK1.1 (clone: PK136, BD), and Ly6G (clone: 1A8, BD) in PE. To characterize leukocytes, we used CD45.2 (clone: 104, eBioscience) in eFluor 450; for myeloid cells, we used CD11b (clone: M1/70, BD) in APC-Cy7; and for macrophage specific identification, we used F4/80 (clone: BM8, Biolegend) in PE-Cy7. For purinergic receptor staining, we used P2Y_1_ (clone: E-1, Santa Cruz) and P2X_7_ (clone: 1F11, Biolegend). As appropriate isotype controls, we used mouse IgG2a (Biolegend), mouse IgG1 (BD), and rat IgG2b (Thermo Fisher). Stained cells were analyzed using a BD FACSCanto II flow cytometer.

### RNA isolation and cDNA transcription

RNA isolation was performed with the RNeasy Mini Kit from Qiagen according to manufacturers’ protocol with the optional DNase step. The obtained RNA quantity and purity was accessed via NanoDrop (Thermo Scientific) and diluted to a final concentration of 100ng/μl for prior cDNA transcription. The transcription into cDNA was performed with the high-capacity cDNA reverse transcription kit (Applied Biosystems) according to manufacturers’ protocol using 1μg of the isolated RNA.

### Real-time PCR

Real-time PCR was performed in a 96-well white-colored RT-PCR plate (Bio-Rad), using 10μl ORA 2x TaqMan Master Mix (highQu), 1μl of the target gene primer (Thermo Fisher), 7μl of nuclease-free water, 1μl of housekeeping gene primer (β-Actin), and 1μl of cDNA per reaction. PCR reaction was performed and data was acquired in a CFX96 touch real-time PCR detection system (Bio-Rad). Following primers from Thermo Fisher (FAM-coupled) were used in this manuscript: Arg1 (Mm00475988_m1), TGFβ (Mm01178820_m1), IRF4 (Mm00516431_m1), IL-1β (Mm01336189_m1), TNFα (Mm00443258_m1), NOS2 (Mm00440502_m1), P2Y1 (Mm02619947_s1), P2Y2 (Mm01274120_m1), P2Y4 (Mm00445136_s1), P2Y6 (Mm01275472_m1), P2Y12 (Mm0195043_s1), P2Y13 (Mm01951265_s1), P2Y14 (Mm01952477_s1), A1 (Mm01308023_m1), A2A (Mm00802075_m1), A2B (Mm00839292_m1), A3 (Mm00802076_m1), CD39 (Mm00515447_m1), CD73 (Mm00501910_m1), P2X1 (Mm00435460_m1), P2X2 (Mm00462952_m1), P2X3 (Mm00523699_m1), P2X4 (Mm00501787_m1), P2X5 (Mm00473677_m1), P2X6 (Mm00440591_m1), and P2X7 (Mm01199500_m1). Expression levels below a threshold of 2^-dCT^ = 4*10^-5^ was designated as very low or even questionably expressed (but with still quantifiable CT values above 31) and therefore designated as below threshold (b.t.). Genes with no quantifiable CT values (CT values above 38) were designated as not detectable (n.d.).

### Fluo-4 AM microscopy

The procedure was performed according to the manufacturers’ protocol (Molecular Probes). Briefly, Fluo-4 AM working solution was produced by vortexing 100μl of 100x power load with 10μl of 1000x Fluo-4 AM (Molecular Probes) in a 15-ml Falcon. Afterwards, 10ml of life cell imaging (LCI) solution (Molecular Probes) containing 20mM glucose and 100μl of the anion-transport inhibitor 100x probenecid (Thermo Fisher) was added. Medium from BMDMs was removed and cells were washed once with LCI solution w/o glucose and probenecid. Cells were covered with 2ml Fluo-4 AM working solution and incubated for 30min at 37°C and 5% CO_2_. Afterwards, cells were washed once with LCI w/o glucose and probenecid. For fluorescence detection, the cells were covered with LCI with 20mM glucose. The cells were recorded under a fluorescence microscope in the FITC channel during stimulation with 200μM ADP (Sigma Aldrich). In order to quantify the fluorescence signal, we recorded different wells throughout the whole experiment. Fluorescence intensity mean values of marked spots were calculated by the Zen imaging software. For background suppression, we marked n = 4 rectangular spots without any cells and measured the fluorescence intensity mean value of those spots at set timepoints during the entire experiment (9, 6, and 3 s before ADP administration; directly at the time-point of ADP administration; as well as 3, 6, 9, 12, 15, 18, and 21 s after ADP administration). Additionally, in order to quantify the fluorescence signal, n = 20 rectangular spots containing cells (we made sure that the whole cell fit into the frame) were marked. In accordance with the background suppression spots, the fluorescence intensity mean values of those spots were calculated for the same timepoints set for the background suppression spots in dependence of the ADP administration time-point. The average of the fluorescence intensity mean values of the background suppression spots were subtracted from the corresponding fluorescence intensity mean values of the spots containing cells. The relative fluorescence intensity represents the relative increase of the background corrected fluorescence intensity mean value in relation to its corresponding background corrected fluorescence intensity mean value 9 s before ADP administration gives the relative fluorescence intensity.

### Caspase-1 assay

Caspase-1 activity was accessed using the caspase-1 Glo kit from Promega. All experiments and data acquisition were performed according to the manufacturers protocol. Inflammasome activation experiments were performed as described previously [6]. Briefly, to prime the inflammasome, BMDMs were incubated for different durations with DMEM + 10% FCS + 1% P/S containing 100ng/ml LPS from *E. coli* 055:B5 (Sigma Aldrich). Assembly was achieved by subsequently adding 5mM ATP (Sigma Aldrich) to the BMDMs.

### Western blot

For immunoblot analysis of total cell lysates, cells were washed with PBS and lysed in SDS- and DTT-containing sample buffer. Proteins were separated by SDS-PAGE and transferred to nitrocellulose membranes using standard techniques [7]. Primary antibodies were as follows: anti-P2X7 (clone: EPR24130-77, Abcam), anti-P2Y1 receptor (APR-009, Alomone Labs), and anti-A1 (clone: HA1, Santa Cruz).

### Statistics

The statistical analysis was performed with GraphPad Prism. The expression and fold change of the examined genes were analyzed using an unpaired two-tailed *t*-test. For the statistical analysis of the relative fluorescence intensity, a two-tailed paired *t*-test was performed.

## Results

### Bone marrow-derived macrophages express a unique purinergic receptor profile

**Fig. 1:**
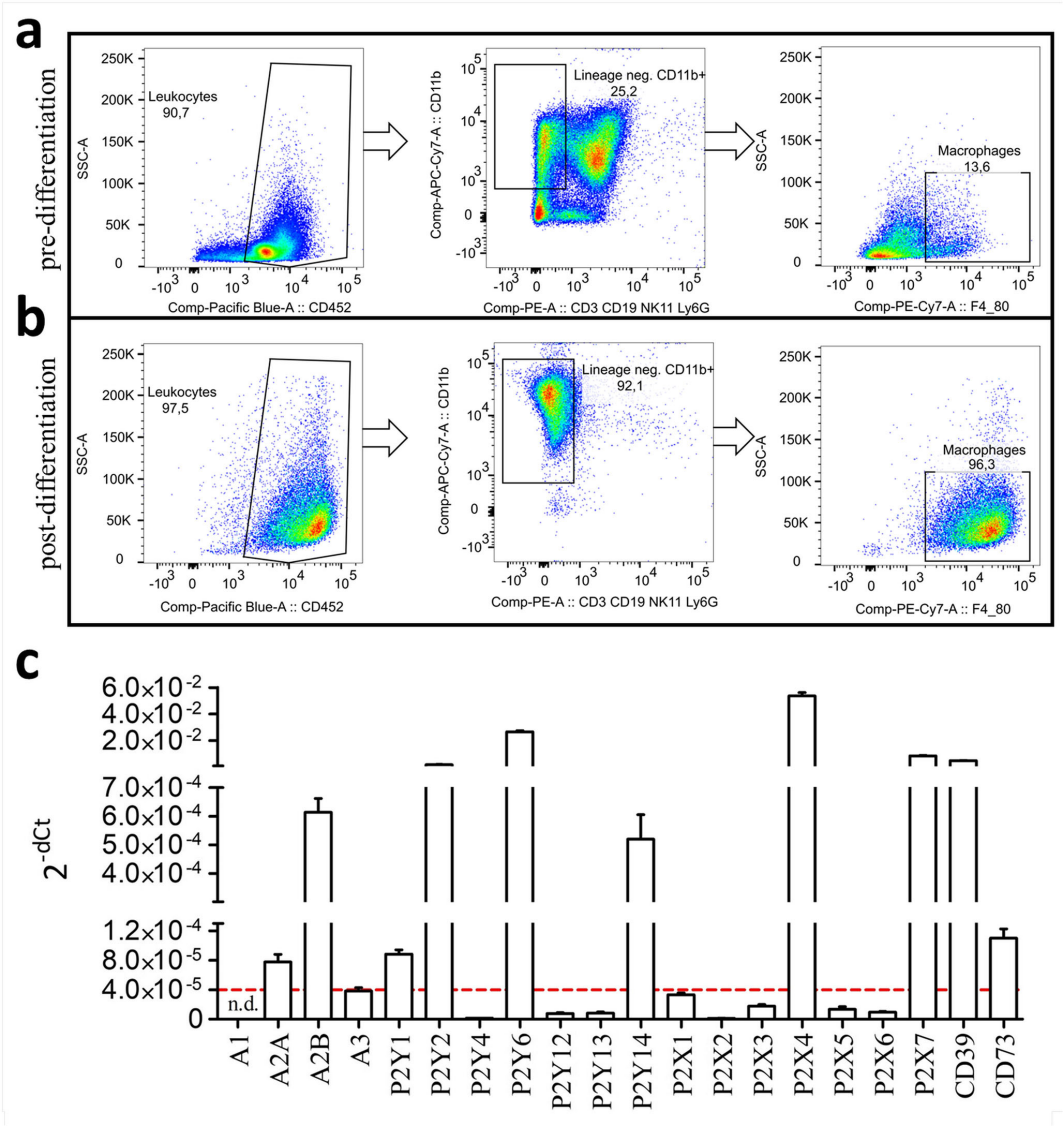
Purinergic receptor expression in bone marrow-derived macrophages (BMDMs) from C57Bl6/J mice. Flow cytometric analysis of **a** directly isolated bone marrow and **b** differentiated 7 days with 30 ng/ml M-CSF. Lineage describes a combination of antibodies recognizing CD3, CD19, Ly6G, and NK1.1, respectively. Macrophages are characterized as CD45^+^, CD11b^+^, and F4/80^+^ and lineage negative population. **c** Real-time PCR purinergic receptor and ectonucleotidase expression profile of unstimulated BMDMs described as 2^-dCt^ values. Red dotted lines visualize the threshold of very low expressional levels. n.d., not detectable

In order to investigate the expression profile of purinergic receptors in macrophages, we cultivated bone marrow cells from C57Bl6/J mice. Flow cytometric analysis of the pre-differentiated, directly isolated bone marrow cell culture revealed a heterogeneous cell suspension consisting of leukocytes (CD45^+^), non-leukocytes (CD45^-^), myeloid (CD11b^+^, lin^-^) as well as other immune cells (lin^+^) with only few CD45^+^, CD11b^+^, lin^-^, and F4/80^+^ macrophages (Fig. 1a). Differentiation of this cell culture via M-CSF resulted in a pure CD45.2^+^, CD11b^+^, lin^-^, and F4/80^+^ macrophage cell culture (Fig. 1b). Expressional analysis of macrophages (CD45^+^, lin^-^, CD11b^+^, F4/80^+^) revealed a distinct purinergic receptor profile (Fig. 1c). We identified high expression of the purinergic receptors P2Y_2_, P2Y_6_, P2X_4_, and P2X_7_. The P1 receptor A2B and P2Y_14_ showed intermediate expression levels, whereas the P1 receptor A2A and P2Y_1_ revealed low expression levels. The ectonucleotidase CD39 was highly expressed, while the ectonucleotidase CD73 showed a low expression. We defined expression levels below a threshold of 4*10^-5^ for the 2^-dCT^ value as very low. This threshold equals a 14.61 dCT value between housekeeping gene and target gene. The CT value of the β-actin housekeeping gene in our experiments was 17.71 ± 1.42 (SD, n=841). Accordingly, we refer target genes with more than 30.9 CT values as below threshold (b.t.) in the following results.

### M1/M2 polarization leads to upregulation of Gαs-coupled P1 receptors, with high expression of the high-sensitivity A2A receptor on M1 macrophages

**Fig. 2:**
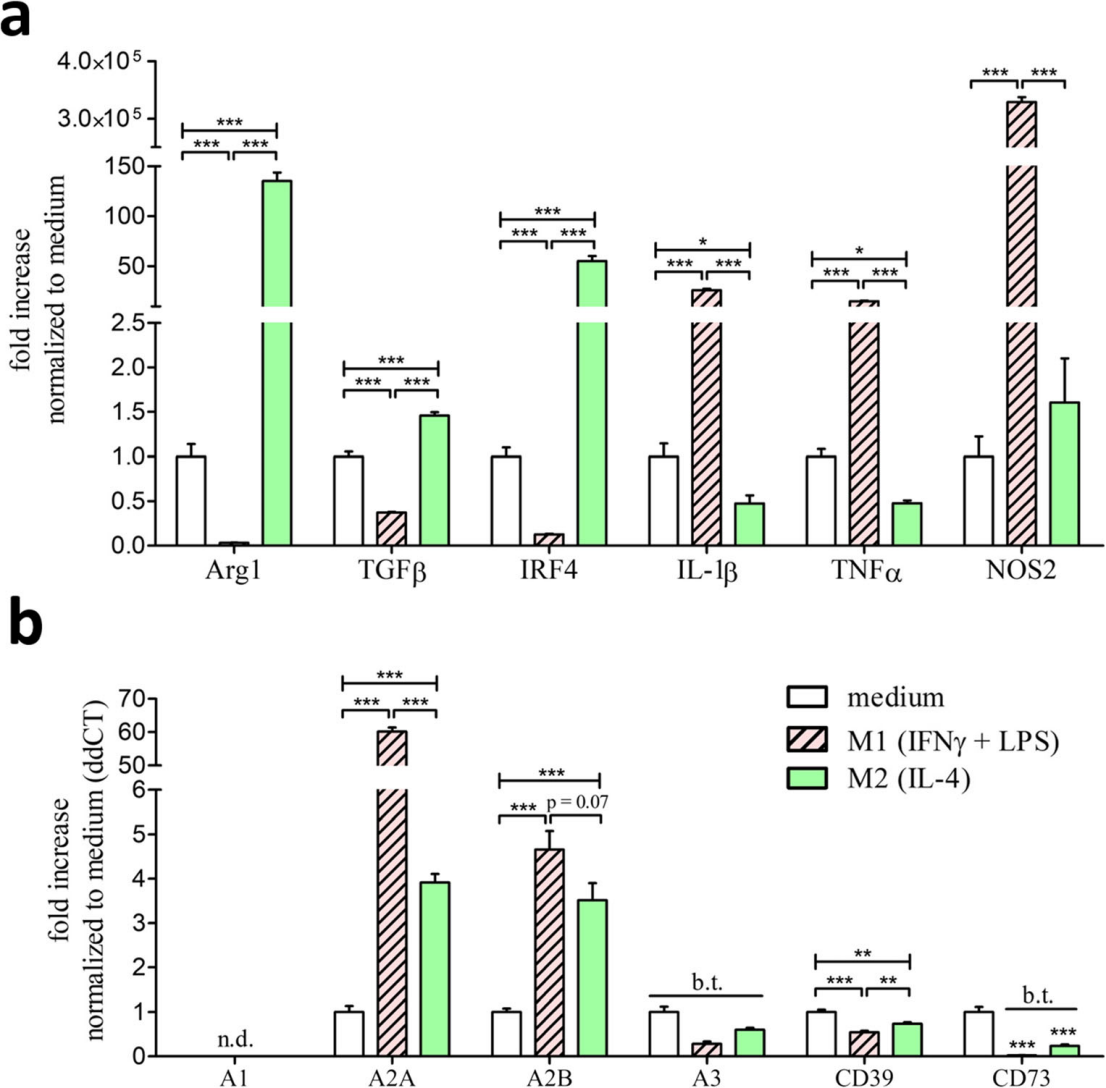
Expression of specific M1/M2 markers upon pro- and anti-inflammatory differentiation as well as P1 and ectonucleotidases in unstimulated M0 compared to differentiated M1 and M2 macrophages. **a** Real-time PCR detection described as ddCt fold increase of specific M1/M2 markers in BMDMs stimulated for 5 h with 10ng/ml IFNγ + 100 ng/ml LPS (red, striped bars) for M1 differentiation and 5 h with 20ng/ml IL-4 (green bars) for M2 differentiation. BMDMs which only received medium for 5 h (white bars) are described as M0 macrophages and serve as reference value for the ddCt fold increase comparison. **b** Real-time PCR expression described as ddCt fold increase referred to M0 expression (white bars) compared to M1 (red, striped bars)- and M2 (green bars)-differentiated macrophages. n.d., not detectable; b.t., below threshold; * p < 0.05, ** p < 0.01, *** p < 0.001

Macrophages can be differentiated into pro-inflammatory M1 and anti-inflammatory M2 macrophage subtypes according to either pro- or anti-inflammatory stimuli [3]. To investigate if these states of differentiation alter the expression of purinergic receptors, we cultivated macrophages and stimulated these with either IFNγ and LPS to promote M1-differentiation or IL-4 to promote M2-differentiation. The efficacy of differentiation was analyzed via the established M2 markers arginase-1 (Arg1), transforming growth factor beta (TGFβ), and interferon regulatory factor 4 (Irf4) as well as the M1 markers IL-1β, tumor necrosis factor α (TNFα), and nitric oxide synthase 2 (Nos2) (Fig. 2a). Stimulation with IL-4 leads to the upregulation of the M2 specific markers Arg1, TGFβ, and IRF4 as well as the downregulation of the pro-inflammatory M1 markers IL-1β and TNFα. Stimulation with IFNγ and LPS leads to the downregulation of the M2 markers and promoted the upregulation of the M1-specific markers IL-1β, TNFα, and NOS2. In the next step, we analyzed the P1 and ectonucleotidase expression in those differentiated M1 and M2 macrophages compared to unstimulated M0 macrophages. Both M1 and M2 differentiation correlated with an upregulation of the Gαs-coupled P1 receptors A2A and A2B (Fig. 2b). In this context, the high-sensitivity adenosine receptor A2A showed a 15-times higher expression in M1 macrophages compared to M2 macrophages. The expression levels of the low-sensitivity adenosine receptor A2B showed no significant difference between M1 and M2 macrophages. Regarding the Gαi-coupled P1 receptors, the high-sensitivity adenosine receptor A1 was not detectable (confirmed by western blot analysis; data not shown) and the low-sensitivity adenosine receptor A3 was below threshold in all macrophage subtypes. Both M1 and M2 differentiation lead to the downregulation of the ectonucleotidase CD39. Nevertheless, M2 macrophages still showed a higher CD39 expression compared to M1 macrophages. The expression of the ectonucleotidase CD73 was below threshold after M1 and M2 polarization.

### M1 polarization leads to the downregulation of the inflammasome regulator P2X_7_ which prevents caspase-1 activation in macrophages after prolonged pro-inflammatory stimulation

**Fig. 3:**
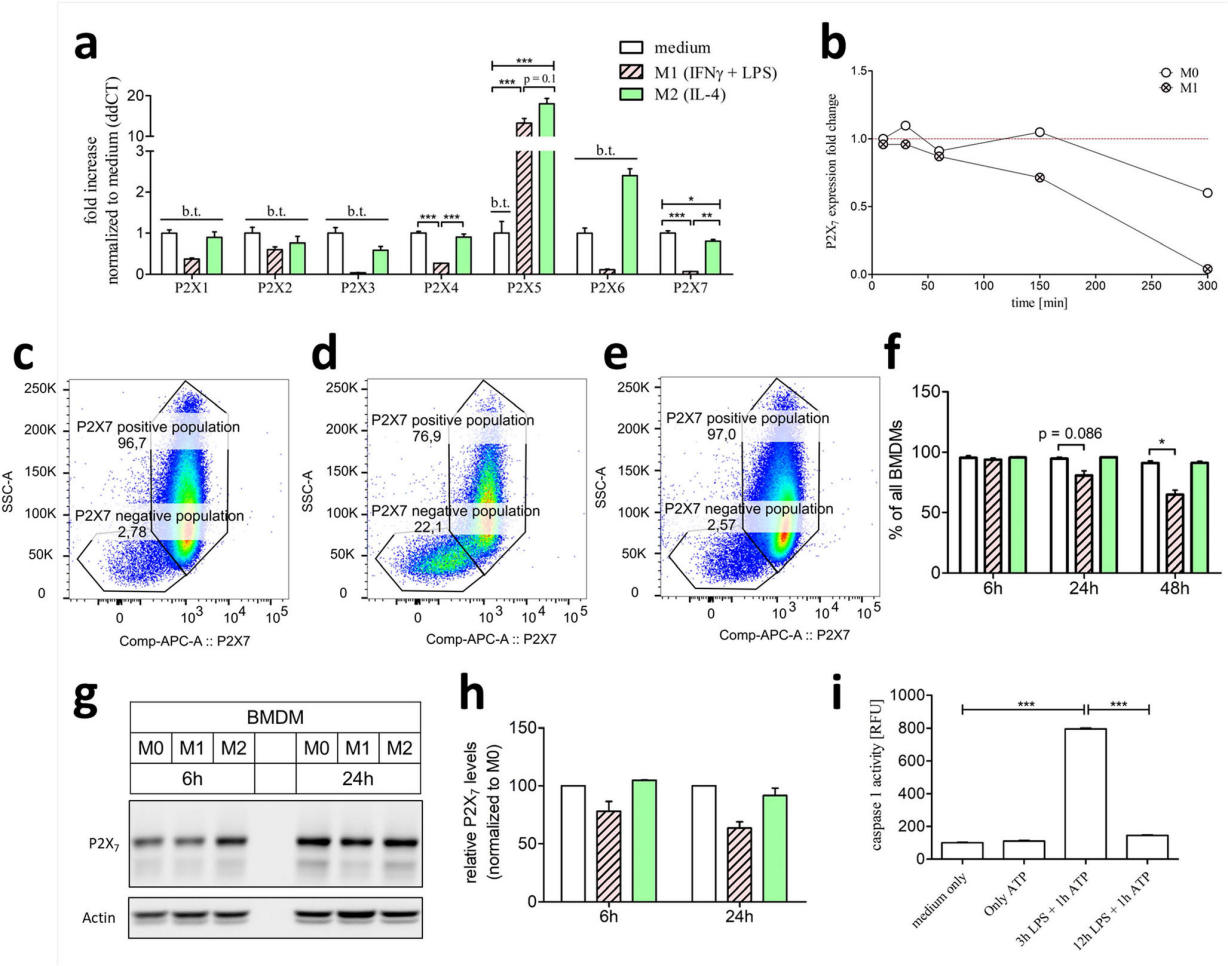
Ionotropic P2X expression in M1-/M2-differentiated macrophages compared to unstimulated M0 macrophages and the impact on inflammasome activation. BMDMs stimulated for 5 h with 10ng/ml IFNγ + 100 ng/ml LPS are described as M1 macrophages (red, striped bars) and BMDMs simulated for 5 h with 20ng/ml IL-4 are described as M2 macrophages (green bars). BMDMs which only received medium for 5 h (white bars) are described as M0 macrophages. **a** Real-time PCR expression of P2X purinergic receptors in M1-/M2-differentiated macrophages described as ddCt fold increase referred to M0 expression levels. **b** P2X_7_ ddCt fold change expression levels over time compared to P2X_7_ expression at time-point t = 0min in M0 macrophages. **c–e** representative flow cytometry dot-blots of 48h stimulated **c** M0, **d** M1, and **e** M2 macrophages. **f** Quantification of the P2X_7_-positive population in percentage of all BMDMs. **g** Western blot analysis of 6h and 24h differentiated M0, M1, and M2 macrophages with **h** normalized quantification to β-actin and in relation to M0 macrophages. **i** Inflammasome activation measured by caspase-1 Glo assay in short-time (3h) and long-time (12h) 100ng/ml LPS primed macrophages and subsequent 5 mM ATP stimulation. b.t., below threshold; * p < 0.05, ** p < 0.01, *** p < 0.001

Naïve M0 macrophages only express the ATP dependent ionotropic receptors P2X_4_ and P2X_7_. Both M1 and M2 differentiation lead to the upregulation of P2X_5_ receptor (Fig. 3a). However, M1 polarization caused a downregulation of P2X_4_ and P2X_7_. Given the pivotal role of P2X_7_ during inflammation via an assembly of the inflammasome [6], it seemed unlikely that this receptor was downregulated in the pro-inflammatory M1 subtype. To investigate a potential early upregulation of P2X_7_ during M1 differentiation, its expression was analyzed at early timepoints (Fig. 3b). Nevertheless, P2X_7_ expression declined over the whole M1 differentiation period. To further analyze this expressional downregulation of P2X_7_ on the protein levels, flow cytometric (Fig. 3c–f) and western blot analysis were performed (Fig. 3g–h). Via flow cytometric analysis, BMDMs can be separated into a P2X_7_ positive and a P2X_7_ negative population. Intriguingly, the P2X_7_ negative population is more pronounced in M1-primed macrophages (Fig. 3d) compared to M0- (Fig. 3c) and M2-primed (Fig. 3e) macrophages. In accordance to the increasing P2X_7_ negative population in M1, the proportion of the P2X_7_ positive BMDM population declines over time in the M1-primed culture compared to M0- and M2-primed cultures (Fig. 3f). This decline of the P2X_7_ positive population was also measurable in a western blot analysis of P2X_7_ (Fig. 3 g and h). To assess if the expression translates into a functional outcome, the inflammasome assembly capacity of M1 macrophages over time was analyzed (Fig. 3i). Whereas short-term M1 differentiation via the pro-inflammatory LPS stimulus followed by ATP administration leads to an increased caspase-1 activity, prolonged pro-inflammatory M1 differentiation completely prevented caspase-1 activation upon ATP administration.

### M1 polarization upregulates Gαi, whereas M2 polarization upregulates Gαq-coupled purinergic receptors

**Fig. 4:**
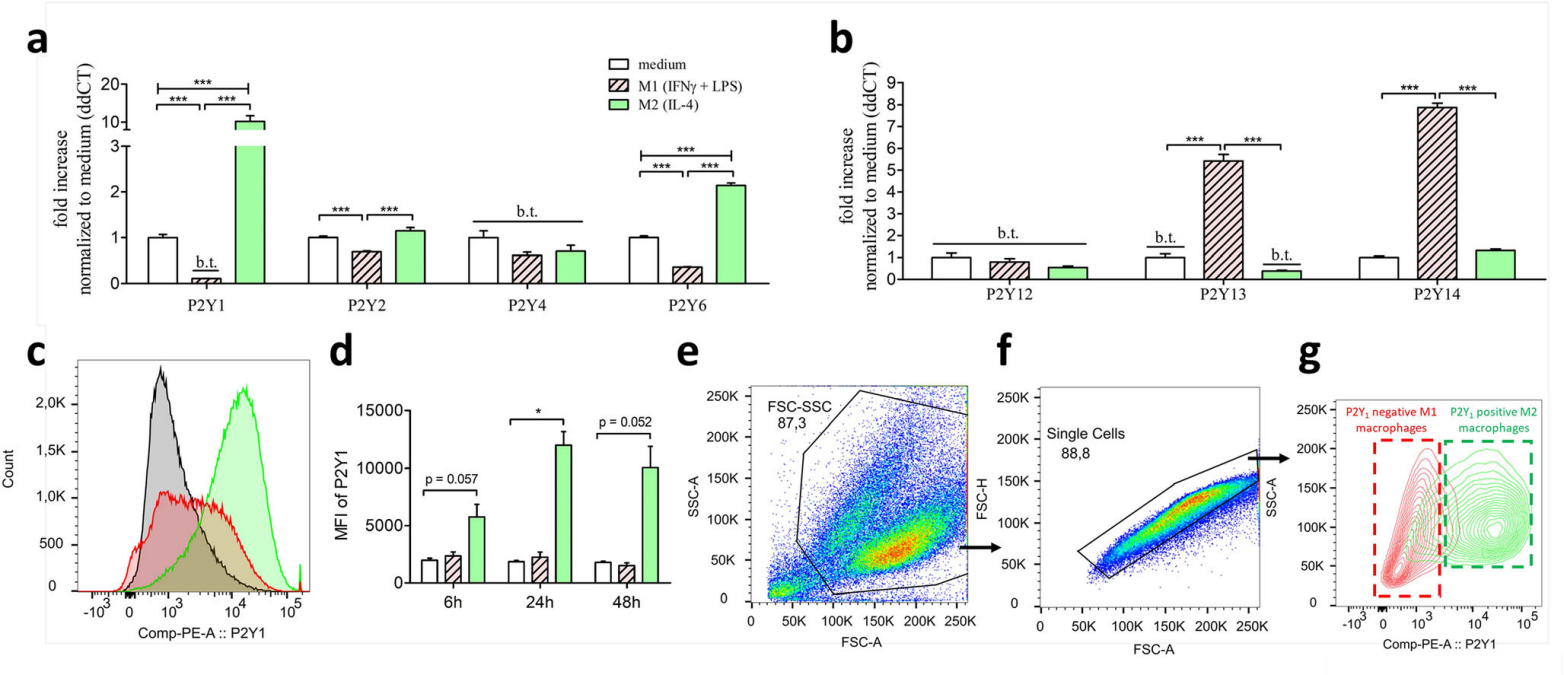
Metabotropic P2Y expression in M1-/M2-differentiated macrophages compared to unstimulated M0 macrophages. BMDMs stimulated for 5 h with 10ng/ml IFNγ + 100 ng/ml LPS are described as M1 (red, striped bars) and BMDMs simulated for 5 h with 20ng/ml IL-4 are described as M2 macrophages (green bars). BMDMs which only received medium for 5 h (white bars) are described as M0 macrophages. **a** Real-time PCR expression of Gq-coupled P2Y purinergic receptors (P2Y_1_, P2Y_2_, P2Y_4_, and P2Y_6_) in M1-/M2-differentiated macrophages described as ddCt fold increase referred to M0 expression levels. **b** Real-time PCR expression of Gi-coupled P2Y purinergic receptors (P2Y_12_, P2Y_13_, and P2Y_14_) in M1-/M2-differentiated macrophages described as ddCt fold increase referred to M0 expression levels. **c** Flow cytometric P2Y_1_-dependent histogram of 24h differentiated M0 (black-lined), M1 (red-lined), and M2 (green-lined) macrophages. **d** Quantification of the P2Y_1_-dependent mean-fluorescence intensity of M0, M1, and M2 macrophages after differentiation timepoints 6h, 24h, and 48h. **e**–**g** Gating strategy of P2Y_1_-dependent M1 and M2 distinction. **e** Debry-exclusion gating via cell size (FSC) and granularity (SSC). **f** Single cell gating via FSC-A (area) and FSC-H (height). **g** M1 and M2 distinction via the cell surface antigen P2Y_1_. b.t., below threshold; *** p < 0.001

The metabotropic purinergic receptors can be divided into Gαq-coupled P2Y_1_, P2Y_2_, P2Y_4_, and P2Y_6_ and Gαi-coupled P2Y_12_, P2Y_13_, and P2Y_14_ receptors, respectively. Whereas M2 polarization leads to the upregulation of the Gαq-coupled ADP receptor P2Y_1_ and the UDP receptor P2Y_6_, pro-inflammatory M1 differentiation promoted the downregulation of the Gαq-coupled P2Y_1_, P2Y_2_, and P2Y_6_ receptors (Fig 4a). Intriguingly, the expression levels of P2Y_1_ decreased below threshold levels in M1 macrophages. The expression levels of the ATP/UTP receptor P2Y_4_ was below threshold in all three macrophage subtypes. However, M1 polarization was accompanied with an upregulation of the Gαi-coupled ADP receptor P2Y_13_ and the UDP-glucose receptor P2Y_14_ (Fig. 4b). Of note, P2Y_13_ was below the detection threshold in both M0 and M2 macrophages, and thereby uniquely expressed in the pro-inflammatory M1 subtype. The expression of the Gαi-coupled ADP receptor P2Y_12_ was below the threshold in all three macrophage activation states. According to the expressional analysis, M2 macrophages could uniquely respond to ADP via activation of the Gαq-coupled P2Y_1_ receptor. Due to this, we aimed to evaluate the P2Y_1_ protein levels in M0, M1, and M2 macrophages using flow cytometric analysis (Fig. 4c–g). Incubation with IL-4 leads to the upregulation of cell surface P2Y_1_ on M2-differentiated macrophages compared to M0- or M1-differentiated macrophages (Fig. 4 c and d). In direct comparison, the P2Y_1_-dependent mean-fluorescence intensity allows the distinction of a P2Y_1_-negative population of M1-differentiated macrophages from a P2Y_1_-positive population of M2-differentiated macrophages (Fig. 4e–g).

### ADP promotes Gαq signaling in M2-polarized macrophages due to a unique purinergic receptor profile

**Fig. 5:**
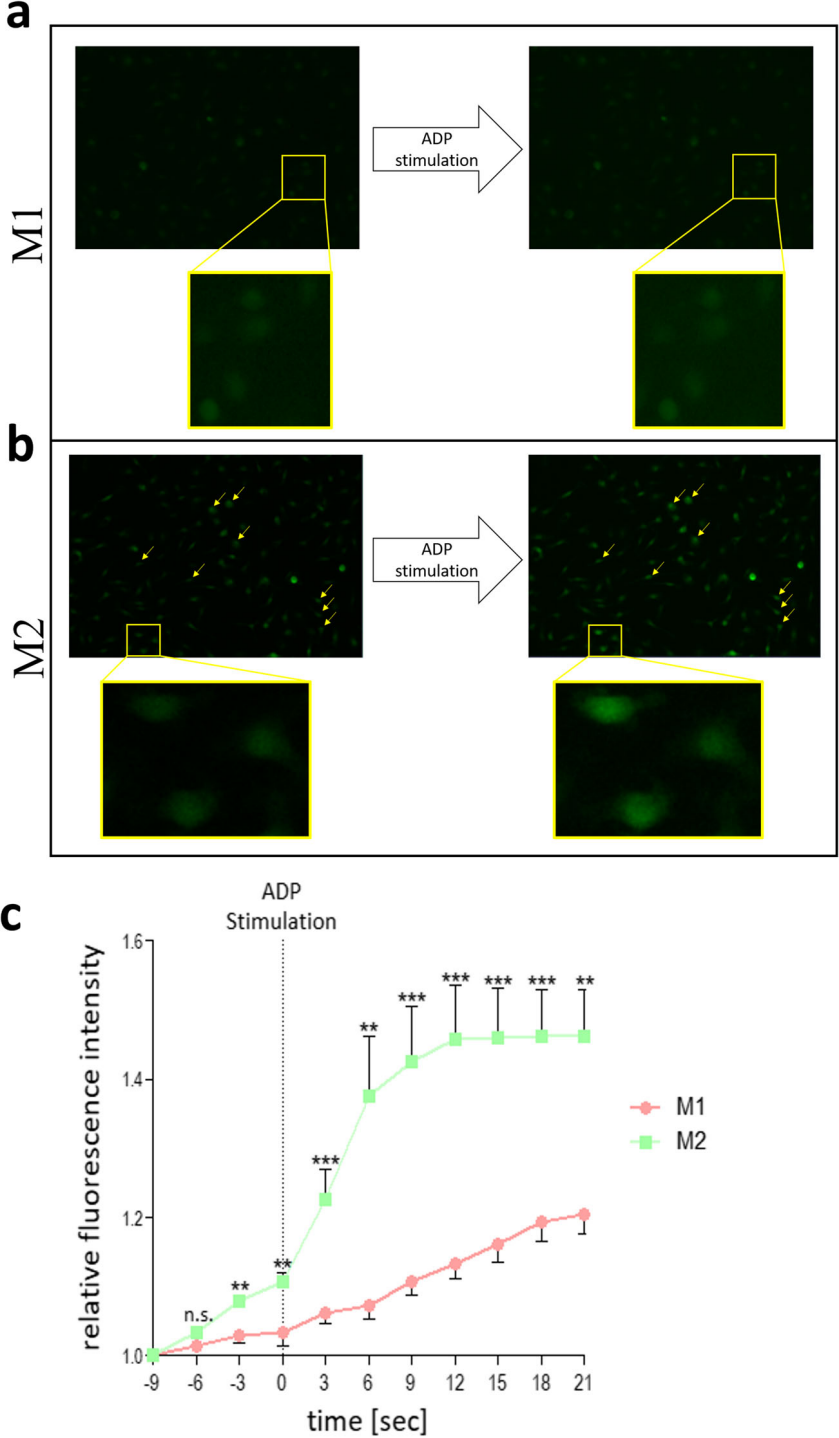
Calcium flux assay in M1 and M2 macrophages upon ADP stimulation. BMDMs stimulated for 5 h with 10ng/ml IFNγ + 100 ng/ml LPS are described as M1 (red dots) and BMDMs simulated for 5 h with 20ng/ml IL-4 are described as M2 macrophages (green dots). Representative pictures of M1 and M2 macrophages loaded with fluo-4 AM **a** prior and **b** after 200 mM ADP stimulation. **c** Quantification of fluo-4 AM fluorescence intensity over time upon 200 mM ADP stimulation in M1 (red dots) and M2 (green dots) macrophages

In order to investigate the functional relevance of the altered expression and protein levels of P2Y_1_, Ca^2+^ flux upon ADP administration was measured in M2-polarized macrophages via Fluo-4 AM. Stimulation with ADP did not show any related increase of the Ca^2+^ dependent Fluo-4 AM fluorescence signal in M1-polarized macrophages (Fig. 5 a and c). Nevertheless, upon ADP stimulation, M2-polarized macrophages responded with an increase of the Ca^2+^ dependent Fluo-4 AM fluorescence signal (Fig. 5 b and c).

## Discussion

In an immunological context, adenosine P1 receptors and the nucleotide degrading ectonucleotidases CD39 and CD73 promote anti-inflammatory functions [8], whereas P2Y and P2X receptors orchestrate immunological processes in a rather pro-inflammatory context. For example, P2Y2 activation is accompanied with increased leukocyte infiltration during smoke-induced lung injury [9], peritonitis [10], atherosclerosis [11], and metabolic syndrome [12]. Depletion of the P2Y2 receptor reduces macrophage accumulation in visceral adipose tissue and ameliorates the outcome of metabolic syndrome in mice [12]. Furthermore, activation of the ionotropic P2X7 receptor leads to the assembly of inflammasome subunits in macrophages. The assembled inflammasome complex activates pro-caspase-1 by autocleavage process to fully functional caspase-1. Activated caspase-1 then proteolytically cleaves and thereby activates pro-inflammatory cytokines like IL-1β. Depletion of P2X7 prevents inflammasome activation in lesional macrophages and improves atherosclerosis outcome in mice [13].

An abundance of immunological processes are regulated by the composition of extracellular nucleotides. Recognition of this nucleotide composition by numerous purinergic receptors further enhances the complexity of this system. In this context, the expression of a certain purinergic receptor repertoire critically defines the way a cell reacts to extracellular nucleotides. In this study, we show that bone marrow-derived macrophages express a unique repertoire of purinergic receptors. Moreover, we show for the first time that this purinergic receptor repertoire is highly flexible and quickly adapts upon pro- and anti-inflammatory macrophage differentiation with functional consequences to nucleotide stimulation.

Unstimulated BMDMs express both the high-sensitivity (A2A) as well as the low-sensitivity (A2B) Gαs-coupled adenosine P1 receptor. The expression levels of the Gαi-coupled adenosine receptors A1 and A3 were either very low (A3) or even not detectable (A1). These observations are in agreement with previous studies which detected A2A, A2B, and A3 on freshly isolated peritoneal macrophages [14]. Both pro- and anti-inflammatory BMDM differentiation leads to the upregulation of A2A and A2B. These findings are consistent with previous findings in which pro-inflammatory stimuli like TNFα lead to an upregulation of A2A in macrophages [9]. For the high-sensitivity receptor A2A, this effect was more pronounced in pro-inflammatory differentiated M1 compared to anti-inflammatory differentiated M2 macrophages (Fig. 2b). There is an evidence that the anti-inflammatory capacity of A2A exceeds the capacity of A2B [10, 11] and that the anti-inflammatory effect of adenosine is mainly driven by A2A activation [12]. Previous studies already described a pivotal role of A2A in dampening the inflammatory capacity of macrophages [13]. Due to this, the observed increased expression of A2A in M1 macrophages could represent a negative feedback loop by an increased susceptibility to adenosine-dependent anti-inflammatory stimuli. The ectonucleotidases CD39 and CD73 degrade extracellular adenine-nucleotides to adenosine and thereby convert P2 signals to anti-inflammatory P1 signaling. Previous studies already described the expression of these ectonucleotidases in freshly isolated macrophages [15]. In this study, we highlighted that compared to M1 macrophages, anti-inflammatory M2 macrophages express higher levels of these ectonucleotidases. By assuming a functional correlation of this expression data, M2 macrophages are more effective in anti-inflammatory P2 to P1 conversion compared to pro-inflammatory M1 macrophages.

Macrophages express a unique repertoire of ATP-dependent ionotropic P2X receptors. Freshly isolated murine macrophages [15] as well as human monocytes [16] express P2X_1_, P2X_4_, as well as P2X_7_. Other investigators observed additional P2X_5_ expression in macrophages derived from peripheral blood monocytes [17] and alveolar macrophages [18]. These observations are in agreement with our current findings of P2X_4_ and P2X_7_ expression in unstimulated BMDMs (Fig. 1C). The expression of the ionotropic P2X_1_ receptor was very low compared to P2X_4_ and P2X_7_ and therefore designated as below threshold in this study. Our study demonstrated for the first time a dramatic downregulation of both P2X_4_ and P2X_7_ upon pro-inflammatory stimulation to M1 macrophages. Flow cytometric analysis revealed a P2X_7_ positive as well as negative population in BMDMs. Intriguingly, only M1-differentiated macrophages experience a decrease of the P2X_7_-positive population over time (Fig. 3c–f). The downregulation of P2X_7_ as well as the reduction of the P2X_7_ positive population was accompanied by a missing ATP-dependent inflammasome activation, which mimics previous studies where missing P2X_7_ signaling completely blocked ATP-dependent inflammasome activation in BMDMs [6]. Activation of the P2X_4_ receptor regulates P2X_7_ open probability but its deficiency failed to block inflammasome activation [19]. The downregulation of P2X_7_ could represent another anti-inflammatory negative feedback loop comparable to the upregulation of A2A in inflammatory M1 macrophages. This effect could counteract excessive inflammasome activation and probably protects the body from overwhelming pro-inflammatory stimuli. In consequence, only non-inflammatory primed macrophages would be capable of appropriate inflammasome activation. However, a prolonged exposure to a pro-inflammatory environment reduces macrophages ATP-dependent inflammasome-activating abilities. Another possible reason for the downregulation of the P2X_7_ receptor could be a mechanism to preserve the viability of immune cells. Activation of the ionotropic P2X_7_ leads to a strong ion-flux and thereby promotes cell death. The downregulation of the death receptor P2X_7_ could prevent cell death and thereby prolong the activity of macrophages in a pro-inflammatory environment. Furthermore, we observed a strong up-regulation of the P2X_5_ in both pro- and anti-inflammatory differentiated macrophages (Fig. 3a). Unfortunately, the detailed function of this receptor on macrophages is mainly unknown but recent investigations point at a potential involvement of this receptor in inflammasome activation [20]. We postulate that immunity against *Listeria monocytogenes* depends on P2X_5_-dependent inflammasome activation. Intriguingly, killing of *L. monocytogenes* was independent of the P2X_5_ ligand ATP, pointing at an alternative currently unknown activation mechanism of the P2X_5_-inflammasome axis. The fact that in our study both M1 and M2 macrophages upregulate P2X_5_ could make them accessible for this ATP-independent alternative inflammasome axis.

Unlike the P1 and P2X, current literature is inconsistent regarding P2Y receptor expression in macrophages. Our investigations revealed the expression of the Gαq-coupled P2Y_1_, P2Y_2_, and P2Y_6_ as well as the Gαi-coupled P2Y_14_ receptor (Fig. 4). Pro- and anti-inflammatory differentiation of macrophages resulted in a clear Gαq/Gαi pattern. Whereas anti-inflammatory M2 macrophages predominantly upregulated Gαq-coupled P2Y receptors, pro-inflammatory M1 macrophages not only upregulated Gαi-coupled but also downregulated Gαq-coupled P2Y receptors. Intriguingly, M1 and M2 macrophages revealed completely contrasting ADP-dependent purinergic receptors P2Y_1_ and P2Y_13_. We detected P2Y_1_ as the predominantly expressed ADP-receptor on M2 macrophages, whereas M1 macrophages exclusively express the ADP receptor P2Y_13_. Additionally, the ADP receptor P2Y_1_ served as a strong marker to distinguish P2Y_1_-negative M1 from P2Y_1_-positive M2 macrophages via flow cytometric analysis (Fig. 4c–g). This contrasting effect suggests further investigations evaluating P2Y_1_ and P2Y_13_ as potential novel markers in order to distinguish between different macrophages subtypes in vitro and in vivo.

The current study highlights for the first time a complete analysis of the flexible nature of the purinergic receptor profile in different macrophage subtypes like M1 and M2. The unique expression profile explains the silencing of ATP-dependent inflammasome activation in M1 macrophages and the ADP-dependent calcium influx in M2 macrophages. Of note, the dichotomous classification of M1 and M2 is a simplification, and macrophage polarization is rather a complex spectrum between different polarization states [2]. Nevertheless, there is strong evidence that macrophages shift between a pro-inflammatory and anti-inflammatory state, and IFNγ with LPS and IL-4 are established stimuli to promote such polarizations. The context-dependent polarization upon environmental stimuli also alters the way macrophages interact with and sense their current surrounding. For example, pro-inflammatory M1 macrophages increase toll-like receptor (TLR) signaling [21] via certain TNFR-associated factors (TRAFs) [22] and upregulate genes involved in endocytosis [3]. The anti-inflammatory M2 polarization upregulates genes associated with lipoprotein uptake [23], fatty acid catabolism [24], and tissue repair [3].

Our study shows that macrophage polarization is accompanied with a unique purinergic receptor profile which renders their behavior towards extracellular nucleotides. This study highlights for the first time the potential of purinergic receptors like P2X_7_ and P2Y_1_ as novel macrophage polarization markers and thereby provides new insights into the role of purinergic receptors to shape the complex function of different macrophage subunits. Future studies will evaluate if the targeting of specific macrophage subsets by interfering with their unique purinergic receptor profile has an impact on inflammatory diseases.
